# Skin lipids alone enable conspecific tracking in an invasive reptile, the Argentine black and white tegu lizard (*Salvator merianae*)

**DOI:** 10.1371/journal.pone.0293591

**Published:** 2023-10-31

**Authors:** M. Rockwell Parker, Eric A. Tillman, Lauren A. Nazarian, Megan L. Barlowe, Julianna M. Lincoln, Bryan M. Kluever

**Affiliations:** 1 Department of Biology, James Madison University, Harrisonburg, VA, United States of America; 2 National Wildlife Research Center, Florida Field Station, Wildlife Services, U.S. Department of Agriculture, Gainesville, FL, United States of America; Instituto Federal de Educacao Ciencia e Tecnologia Goiano - Campus Urutai, BRAZIL

## Abstract

Locating potential mates in non-native habitats is one of the most important challenges faced by invasive vertebrate species. The Argentine black and white tegu lizard (*Salvator merianae*) is a major invasive reptile species in the contiguous United States and is rapidly expanding its range across Florida and the Southeast, in part due to inadequate management strategies and tools. Because a wide array of reptiles, especially squamates (snakes and lizards), have been well-studied for their reliance on chemical cues to locate conspecifics, our project aimed to isolate chemical cues from tegus and assess the ability of adult males and females to use only these cues to make navigation decisions within a Y-maze. Previously, we found that both males and females can follow conspecific scent trails, but the nature of the specific cues used by the tegus was unknown. In this study, we extracted skin lipids from male and female shed skins acquired during the breeding season then tested the extracts for bioactivity at different dilutions prior to Y-maze trials. Both sexes showed positive reactions (e.g., tongue-flicking, nose taps, scratching) to 2:1 skin lipid:hexane dilutions. In the Y-maze, males (n = 7) and females (n = 7) were run in three types of trailing scenarios with these skin lipid extracts: Male-only (pooled lipid extracts from male shed skins), Female-only (extracts from female shed skins), and Male vs. female. Regardless of the tegu lipid type present, tegus preferred to follow the conspecific lipid trail when paired with a neutral control (peanut oil; 2:1 dilution). They also preferred opposite-sex skin lipid trails when paired with same-sex skin lipid trails. We analyzed our results further by comparing them to those of Richard, Bukovich, et al. (2020). We found a synchronization effect of the skin lipids: sex differences in behavior detectable in the scent trail trials were absent when only conspecific skin lipids were present in the environment. Our results indicate that skin lipids alone are sufficient to facilitate mate tracking in *S*. *merianae*, these chemical cues induce reliable behavior, and extracted skin lipids have potential for modifying movement patterns of an invasive reptile in their non-native range. If leveraged in concert with current tegu management strategies (i.e., trapping), extracted skin lipids have the potential to bolster management efficacy but field trials are a requisite next step.

## Introduction

Mate searching in vertebrates requires sex-specific cues that receivers can detect and follow in diverse habitats [1]. Many sexual signals enable mate tracking, but across long distances and/or complex habitat types, few signaling modalities preserve signaler information and persist within the environment due to the significant physical constraints therein [2, 3]. For example, many acoustic signals are information-rich and can be transmitted significant distances, but their quality is environmentally dependent and subject to rapid decay (e.g., [4]). Indeed, many bird species have developed receptive mechanisms for interpreting this decay to identify and locate mates across diverse habitats at significant distances (ranging hypothesis; reviewed in [5]). These types of signals require both vigilant emission and perception to be effective. On the contrary, chemical signals such as pheromones can transmit valuable information while being relatively labile across a range of environments and abiotic conditions (reviewed in [6]). For example, territorial markings made by mammals are deposited in a given location and will remain useful sources of signaler information without the signaler being present or constantly reproducing the marks (reviewed in [3]).

Squamate reptiles (snakes, lizards) use a diversity of signaling modalities to locate and assess potential mates, but chemical signals are the predominant modality common to all species that have been tested [7]. Some squamate chemical signals are actively deposited as territorial marks (i.e., in many *Sceloporus* species), and receivers assess these signals to determine various qualities about the signaler (e.g., reproductive condition, mate quality; reviewed in [8]). Other species create chemical trails passively as they navigate environments during the breeding season which are then used by receivers to track potential mates and assess them. This is a predominant mechanism in snakes [9]. One example is the red-sided garter snake (*Thamnophis sirtalis parietalis*), where females produce a sex pheromone that encodes information about female size, condition, and species and serves as a chemical trail used by males to track mates during the breeding season (e.g., [10, 11]).

Despite the fact that many reptile species are broadly successful at invading non-native habitats and that reptiles are adept at using chemical cues to locate mates, relatively little is known about the chemical ecology of invasive reptiles. The brown treesnake (*Boiga irregularis*) is the only invasive reptile for which a significant number of studies have evaluated their reliance on chemical cues in mate choice; but, those studies collectively indicate that sexual chemical cues are not key to mate tracking [12]. And while work is currently assessing the role of reproductive chemical cues in another invasive snake, the Burmese python (*Python bivittatus*), nothing is known about their chemical ecology in the endemic range [13, 14]. Significant effort has been required to parse the reproductive chemical ecology of invasive vertebrates when their endemic chemical ecology is unknown. For example, the sea lamprey (*Petromyzon marinus*) remains an enigmatic invasive vertebrate in the Great Lakes region of the United States that has been studied most extensively in non-native habitats [15]. As a result of intense research drive to inform management practices on sea lamprey, an array of discoveries revealed that specific chemicals act on individual components of the male-female reproductive dynamic: blends of some compounds activate spawning while others facilitate mate tracking. These studies pinpointed the cues selected for development as chemical lures that have enhanced trapping and removal efforts over the last 30 years [15]. Therefore, studies targeting reproductive chemical cues as management tools in other vertebrates have significant potential, especially if a given species has been studied in its native range.

The Argentine black and white tegu (*Salvator merianae*, hereafter tegu) is a major invasive species of concern that demonstrates a reliance on chemical cues during mate searching. As predacious omnivores, tegus pose a significant conservation concern as invasive populations threaten native wildlife, especially ground nesting birds and reptiles which occupy burrows [16–18]. Tegus also disrupt island ecosystems via direct and indirect effects on endemic and endangered species [19, 20]. Owing to their omnivorous diet, invasive populations of tegus represent potential threats to other resources, particularly agricultural industries [21]. The species is also a growing management concern in the southeastern United States due to their broad habitat use and high tolerance to cold temperatures, enabling significant potential for range expansion [22]. Contemporary management tools available to managers, predominantly various configurations of live traps baited with eggs [23], have been reported as being inadequate, with additional methods/tools being needed to protect natural resources from invasive tegus [24].

Tegus, similar to other teiid lizards, rely on chemical cues to track mates [25], and the production of these chemical cues is tied to seasonal reproductive physiology and activity. In both sexes, sex hormones (androgens, estrogens) reach peak circulating levels following brumation in their native range (September-December; South America) [26, 27]. Male tegus exhibit greatest reproductive activity coincident with the increase of testosterone [26]: most importantly, male femoral pore secretions, a major source of chemosignals in many lizard species, also increase at this time [28]. There is also evidence that female tegus produce chemosignals used by males [29].

Recently, our research group reported that both male and female *S*. *merianae* can use scent trails from conspecifics to locate potential mates during the breeding season [25]. We also discovered significant sex differences in chemosensory behaviors, notably that females demonstrated higher precision and faster decision-making than males in the same scent trailing apparatus. Because both sexes of Argentine black and white tegus rely on scent trails to assess and track potential mates, we designed an experiment to isolate the putative source of these cues. Squamate reptiles primarily use skin lipids as sources of conspecific information, and femoral pore secretions in tegus are seasonally variable, thus we hypothesized that skin lipids alone would facilitate mate tracking in *S*. *merianae*. We also expected to see sex differences in behavior and performance similar to what we observed recently, and we compared our results from the current study to those of Richard, Bukovich et al. [25].

## Materials and methods

### Study species and husbandry

Adult male (*n* = 7) and female (*n* = 7) Argentine black and white tegus (*Salvator merianae*) were captured from the wild in the vicinity of Homestead, FL (Miami-Dade County) and transported to the U.S. Department of Agriculture’s Wildlife Services National Wildlife Research Center (NWRC) Florida Field Station (hereafter field station) in Gainesville, Florida, USA. The tegus were adult body size (average male snout-to-vent length [SVL] = 39.8 cm, mass = 2.9 kg; female SVL = 36.7 cm, mass = 2.0 kg) and presumed to be sexually mature (S1 Table). All experimental animals were maintained individually in outdoor enclosures as described elsewhere [25]. Food was provided one to three days each week, depending on animal activity and appetite; water was provided *ad libitum*. Tegus were captured and brought to the field station at different times (2010, 2013, 2018), but all animals were acclimated to captivity and spent at least two winters in the outdoor pens prior to testing. All behavioral trials were conducted in spring at the onset of breeding activity in the enclosures, defined as the time after brumation when a marked increase in basking and pacing activity by both sexes was repeatedly observed.

Tests with skin lipid extracts inside individual enclosures were conducted from 14 Apr 2021 to 19 Apr 2021; Y-maze trials were dependent on the outcomes from the enclosure trials and were thus run from 22 Apr 2021 to 18 May 2021. This temporal window for conducting behavioral trials with Argentine tegus in North Florida is later than the identified breeding season for this species in South Florida (March) [30], but the delay in reproductive timing between these sites is expected given their differences in seasonal climate patterns. Further, comparing the results of these trials to those of Richard, Bukovich, et al. [25] was a secondary goal, thus the timing and design of the experiments was aligned with that study. All behavioral testing methods involving the husbandry and use of live vertebrates were approved by the IACUC of the U.S. Department of Agriculture (study protocol QA-2901), and field collection and housing of wild Argentine black and white tegus was approved by Florida Fish and Wildlife Conservation Commission. All efforts were made to minimize animal suffering.

### Experimental apparatus

**Fig 1:**
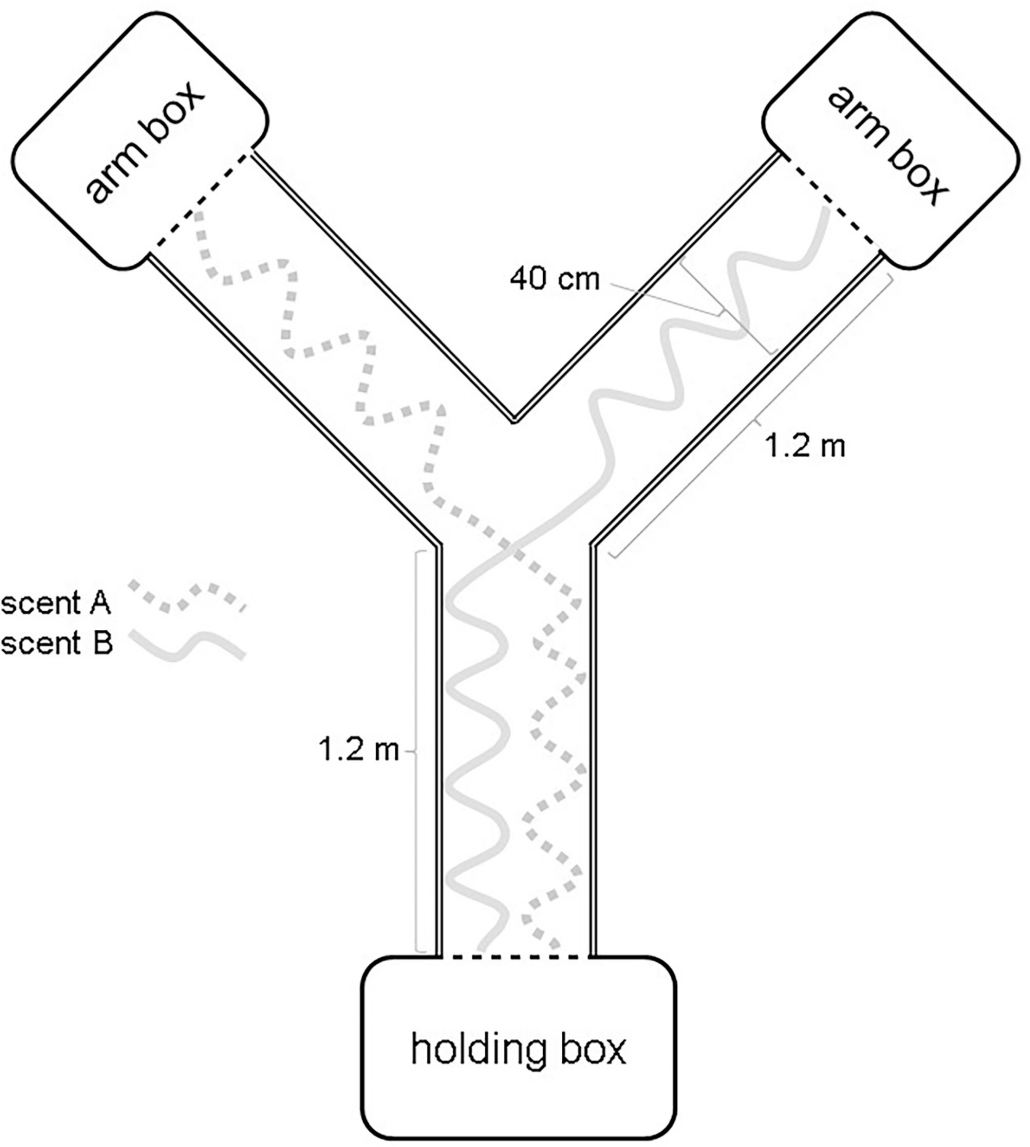
Schematic of experimental lipid trail deposition within a Y-maze. All scent trails started at the opening of the holding box at the base (dashed black line) then run along one side of the arm and crossed to the opposite side, terminating at the opening of an arm box. The trail represented in the schematic (A or B) depended on the specific trial type (i.e., for Male-only trials, male skin lipids would be trail A while peanut oil was trail B). Additional aspects of the Y-maze design can be found in Parker, Currylow, et al. [14].

A Y-maze was used to run all scent trail tests and has been described elsewhere [14, 25]. Briefly, the Y-maze was designed as a fully enclosed testing apparatus fitted with a transparent top for observing tegu behavior in the maze (Fig 1). Stationary cameras enabled network video recording of the tegus across the trials and from multiple angles to quantify behaviors. Three holding boxes were fitted with sliding doors to permit in/egress for a given test tegu, and the holding box at the base of the Y was used as the initial transport and acclimation box.

Trials were conducted between 1000 and 1700 hours. All pieces of the maze (top, sides, and holding boxes) were thoroughly washed with Micro-90® cleaning solution then rinsed with water and air-dried between trials. The base of the apparatus was covered with new plastic sheeting and new paper before starting a trial. Nitrile gloves were worn when washing and assembling the apparatus and boxes to minimize novel odors in the maze.

### Skin lipid extraction

Shed skins from the study tegus (n = 7 males, n = 7 females) were collected over the course of one spring (March to May 2019) from the enclosures when breeding related behavior was observed and maximal. Individual shed skins were allowed to air dry, then were vacuum-sealed and stored at room temperature until lipid extraction. Following an established protocol [31], individual shed skins were cleaned of debris, the cloacal region was removed to avoid contamination with feces or musk secretions, and then the shed was cut into smaller sections (~5 cm^2^) to facilitate extraction. Sheds were extracted in hexane (~500 ml) overnight at room temperature in clean glass beakers. The next day, sheds were removed using metal forceps and the hexane evaporated using a rotary evaporator to obtain skin lipid mass (mg) per shed skin. The extraction was validated by regressing log-transformed skin lipid mass on log-transformed shed skin mass (S1 Fig; S1 Table) [31]. To create a source solution with sufficient lipid for conducting bioassays, extracted lipids were combined per sex to yield a pooled male and pooled female skin lipid extract. Extracts were standardized to the same concentration for testing (10 mg/ml, lipid/hexane) then diluted to 4 mg/ml, 2 mg/ml, and 1 mg/ml for the bioassay prior to Y-maze trials. Peanut oil was chosen as a neutral control scent because of its similar properties to skin lipids and its novelty to the test animals. The peanut oil extracts were prepared at the same concentration as the skin lipid extracts.

### Dilution bioassay

To determine if tegus would demonstrate positive responses to the skin lipids alone, individual tegus were presented with multiple dilutions of the lipid extracts as well as two controls (peanut oil in hexane [1 mg/ml], hexane only). A piece of clean butcher paper was attached to a board, and 0.25 ml of a given solution was applied to a specific area along the paper. Four solutions were presented at once per paper, and the two controls were always included along with randomly selected dilutions of either male or female skin lipids.

Male (*n* = 4) and female (*n* = 4) tegus were presented with a scent board in their individual enclosures and their responses video recorded. Trials were started once tegus had emerged from their overwintering boxes and demonstrated increased activity in April. Boards were placed directly in front of tegus and then observed for 30 min.

Tegus demonstrated several behaviors indicative of chemosensory stimulation and behavioral activation that were previously identified [25]: tongue-flicking, nose-tapping, and scratching. There were no clear sex differences in behavior and the 2 mg/ml skin lipid extracts elicited the most consistent responses. Based on these qualitative results, the 2 mg/ml concentrations of skin lipids were chosen for the Y-maze trials. The peanut oil used as a neutral lipid trail was diluted to the same concentration.

### Trials

Before testing experimental scent trails, bias tests were conducted where no scent was present in the maze. Previous results demonstrated that tegus show no bias for either arm of the apparatus when no scent is present [25].

Tegus were assigned to trial types in a fully randomized design, and each trial allowed a given focal tegu the opportunity to explore the scented maze. Three types of lipid trails were presented to the tegus: Male-only, Female-only, and Male vs. female. The trails were created by drawing 2 ml of solution into a glass pipet, dragging this pipet in a sinuous pattern along one side of the base along the paper substrate, crossing at the junction of the Y to the opposite arm, then continuing the pattern through that arm to the end (Fig 1). Solution was consistently expelled from the pipet to provide only 2 ml of a given scent in the entire maze. For Male- and Female-only trials, peanut oil (2 mg/ml) was used to create a neutral odor trail that ran in parallel with the skin lipid trail in the base before then crossing to the opposite arm (Fig 1). The solutions were overlapped at the junction of the Y to force the tegus to reassess the trails before choosing an arm [9, 14]. The trails were created on the paper and the hexane allowed to evaporate (10 min) before assembling the Y-maze.

Trials were run similarly to Richard, Bukovich et al. [25]. For a single trial, an individual tegu was placed in a holding box and allowed to acclimate for approximately 30 min while the box was attached to the base of the Y. The door to the holding box was then opened, and the focal animal was allowed to move freely throughout the maze. The trial was considered completed when the focal tegu’s head crossed into the holding box at the end of the chosen arm.

### Response variables and behaviors

From recordings of tegu behavior, arm choice, choice penalty score [13, 32], rate of tongue-flicking (tongue-flicks per min), pauses, turns, passes through each arm, and various trailing times were recorded to match Richard, Bukovich, et al. [25]. Choice was scored when the tegu’s head entered the holding box at the end of an arm; behaviors were continually scored for the duration of time a tegu spent in the maze after choice as well. Choice penalty is a negative score based on how far the tegu moves into the non-target arm (peanut oil in the Male-only and Female-only trials; same sex in Male vs. female trials). Rate of tongue-flicking was recorded as the number of visible tongue-flicks per span of time in seconds then converted to tongue-flicks per minute. For all other behaviors, only counts were recorded. Examples of some of these behaviors can be seen in the supplementary video (S2 Fig), and all data are available in S1 Table.

All behavioral variables were assessed in a single temporal context which was until first arm choice was made. Unlike Richard, Bukovich, et al. [25], tegus in the lipid extract trials did not extensively explore the maze after making their first choice.

### Statistical analyses

Binomial tests were used for arm choice data. Two-way (sex, trial type) repeated measures ANOVA followed by pairwise comparisons (Student’s t tests) were used for rate of tongue-flicking, choice penalty score, and all behaviors for both trial phases. Because number of passes through each arm was scored, a separate set of analyses were conducted to assess if tegus differentially investigated the arms of the Y-maze using two-way (arm, trial type), repeated-measures ANOVA followed by pairwise comparisons.

Results from the Y-maze trials were also analyzed to compare behaviors between this experiment and our previous experiment testing tegu-created scent trails [25]. Individual tegu responses were averaged across trial types independently for the scent trail [25] and lipid trail data sets, and response variables were then analyzed using two-way (experiment type, sex), repeated-measures ANOVA.

Statistical significance was set at P < 0.05, and marginal differences were also reported (0.05 < P < 0.1) given that sample size was modest but the experiment had a randomized repeated measures design. All statistical analyses and data visualizations were run in SigmaPlot 13.0 (Systat Software Inc.).

## Results

### Y-maze performance

**Fig 2:**
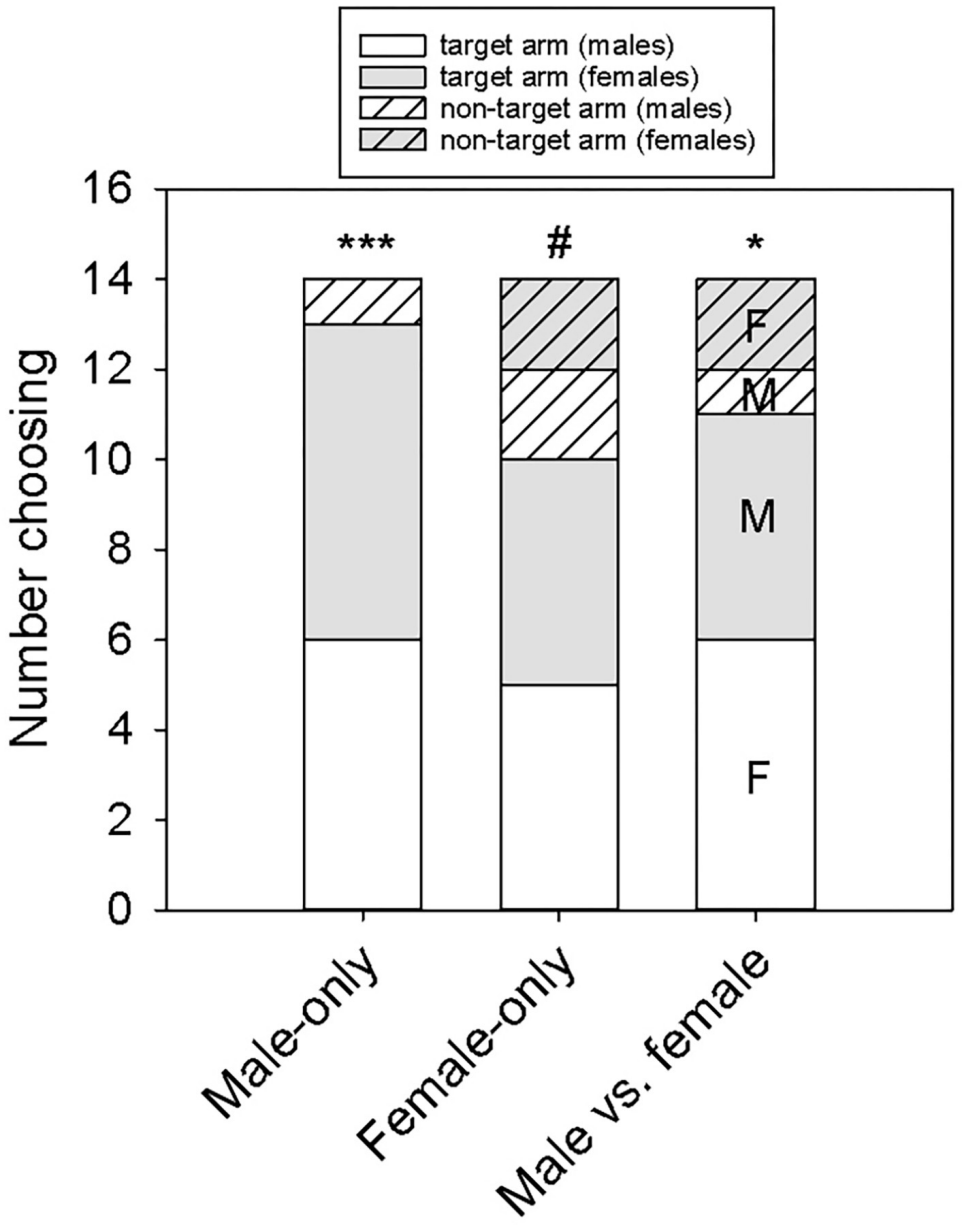
Tegus successfully choose conspecific skin lipid trails in a Y-maze. Tegus (*n* = 7 males, *n* = 7 females) consistently followed isolated skin lipid trails from male conspecifics when only those lipids were present in the base and target arm (Male-only). When presented with a choice between male and female skin lipid trails, tegus chose the opposite-sex trail (target arm) rather than the same-sex trail (non-target; Male vs. female). In Male-only and Female-only trials, the non-target arm contained a neutral lipid trail (peanut oil) at the same concentration as the target lipid trail. ***P < 0.001, *P < 0.05, #0.05 < P < 0.10.

Tegus showed a strong preference for male lipid trails (13/14, P = 0.0009) (Fig 2). Tegus had a marginally significant preference for female lipid trails (10/14, P = 0.06). When presented with both male and female lipid trails simultaneously, tegus chose the lipid trail of the opposite sex (11/14, P = 0.02). Choice penalty scores did not differ due to sex or trial type (sex: F_1,41_ = 0.25, P = 0.62; trial: F_2,41_ = 0.77, P = 0.47; sex × trial interaction: F_2, 41_ = 0.16, P = 0.85) (S3 Fig). Trailing times did not differ due to sex or trial type (sex: F_1,41_ = 0.26, P = 0.61; trial: F_2,41_ = 1.12, P = 0.34; sex × trial interaction: F_2, 41_ = 2.28, P = 0.12) (S4 Fig).

### Behaviors

**Fig 3:**
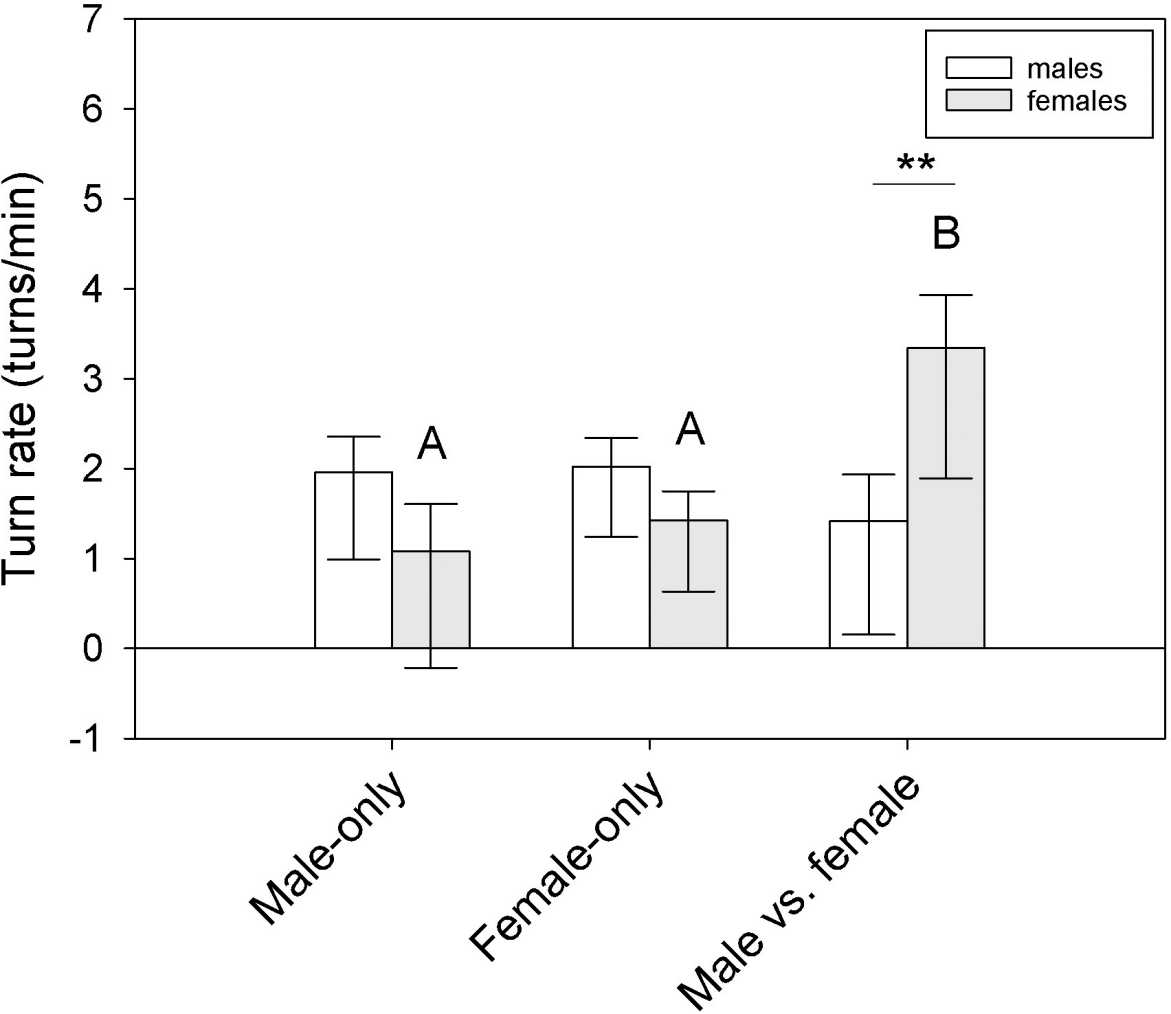
Turn rates in the Y-maze differed between the sexes and across trial type. Female tegus turned more frequently when both male and female skin lipid trails were present, and they turned more than males in that specific type of trial (Male vs. female). Bars are means (+SEM; -95% C.I.). Different letters represent statistically significant differences (P < 0.05) between those trials for a given sex. **P < 0.01.

There was a significant interaction between sex and trial type for turn rate within the Y-maze (F_2,41_ = 7.42, P = 0.003), but no significant independent effects of sex or trial type (sex: F_1,41_ = 0.10, P = 0.74; trial: F_2,41_ = 2.52, P = 0.10) (Fig 3). Females turned more frequently in the Male vs. female trials than both the Male-only (q = 5.64, P = 0.002) and Female-only trials (q = 4.78, P = 0.003; Male-only vs. Female-only: q = 0.86, P = 0.54). Females also turned more frequently than males within the Male vs. female trials (q = 4.19, P = 0.006). No other differences were statistically significant. There were no differences in rates of tongue-flicking (RTF, tongue-flicks per min) due to sex or trial type (sex: F_1,41_ = 0.02, P = 0.87; trial: F_2,41_ = 0.48, P = 0.62; sex × trial interaction: F_2,41_ = 1.91, P = 0.16). There were no differences in pause rates due to sex or trial type (sex: F_1,41_ = 2.87, P = 0.11; trial: F_2,41_ = 0.83, P = 0.44; sex × trial interaction: F_2,41_ = 0.53, P = 0.59).

### Comparing tegu behavior between scent trails and lipid trails

**Fig 4:**
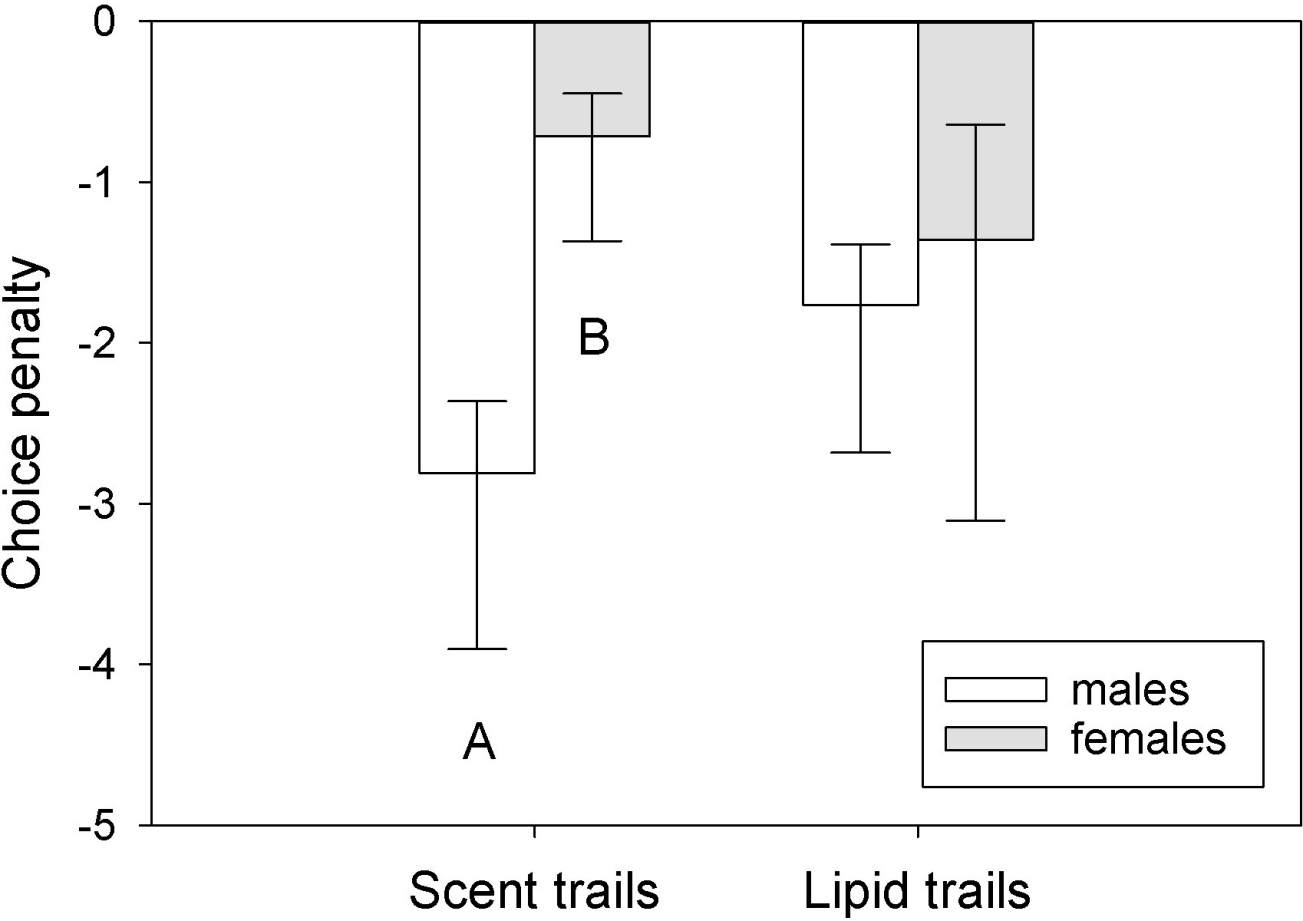
Comparison of choice penalty between trailing scenarios. When tegus created the scent trails tested in the Y-maze [25], there were sex differences in average choice penalty (the degree of exploration of the unscented arm of the maze; 0 to -5 scale) per tegu from the three trial types (Male-only, Female-only, Male vs. female). This sex difference was absent when only skin lipids from conspecifics were present in the maze (Lipid trails). Bars are means (+SEM; -95% C.I.). Different letters represent significant differences (P < 0.05) between those sexes for a given trial type.

**Fig 5:**
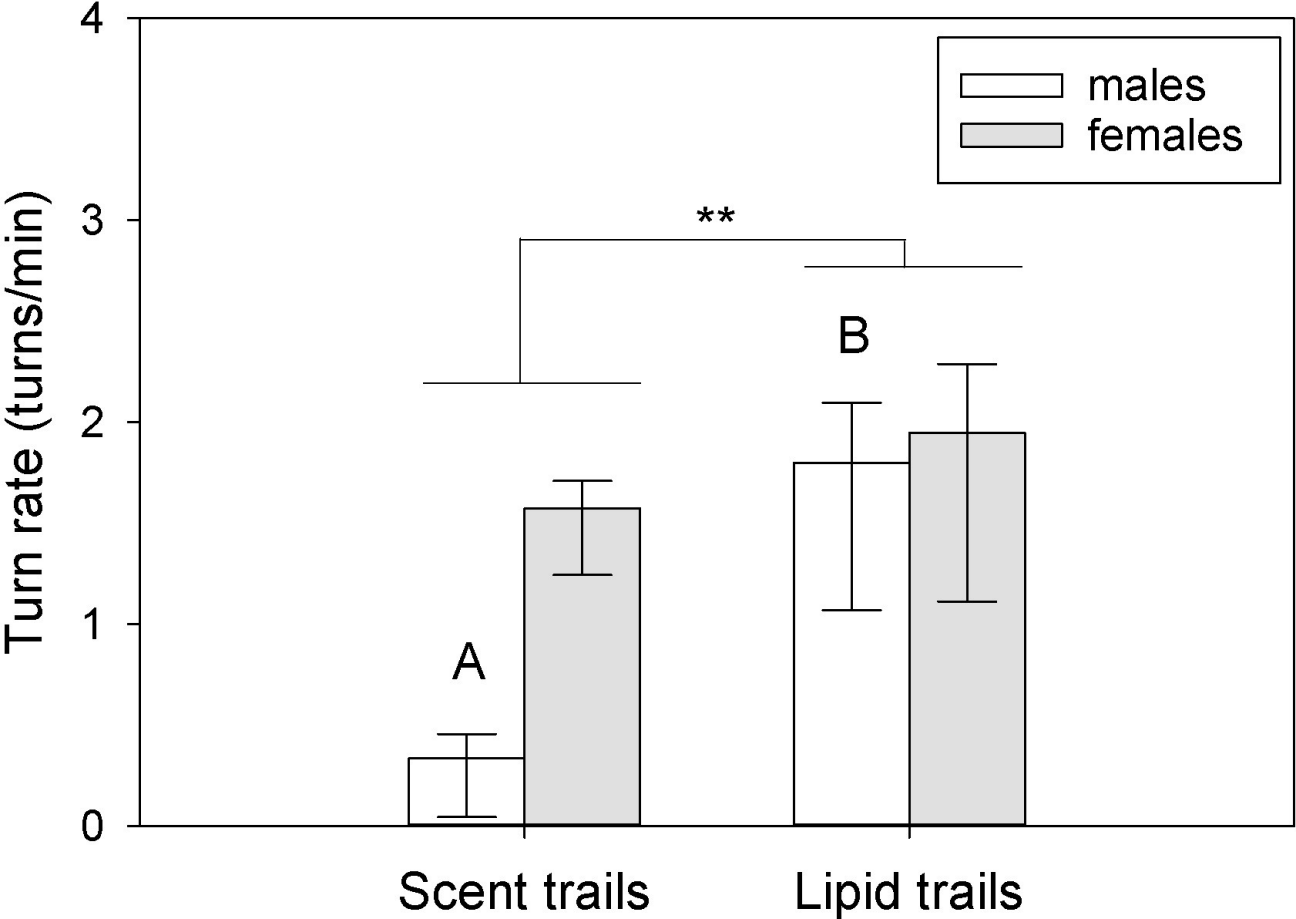
Turn rates differ based on trailing cues. Tegus turned more frequently when only lipid trails were present in the Y-maze, and this was primarily due to the increase in male behavior. Bars are means (+SEM; -95% C.I.). Different letters represent significant differences (P < 0.05) between those trial types. **P < 0.01.

Average choice penalty was influenced by sex (F_1,27_ = 5.42, P = 0.038) but only when tegus were exposed to scent trails (q = 4.36, P = 0.005) and not lipid trails (q = 0.84, P = 0.55) (Fig 4). There was also a marginal sex × trail type interaction (F_1,27_ = 4.10, P = 0.06) but no independent effect of trail type (F_1,27_ = 0.23, P = 0.63). Sex and trail type had significant effects on turn rates (sex: F_1,27_ = 7.15, P = 0.02; trail type: F_1,27_ = 16.32, P = 0.002), and there was a significant sex × trail type interaction (F_1,27_ = 5.73, P = 0.034) (Fig 5). Males turned more frequently in the maze when lipid trails were present (q = 6.43, P < 0.001); females turned more frequently than males while following scent trails (q = 5.07, P = 0.002) but not lipid trails (q = 0.61, P = 0.67). No other differences were significant. For rates of tongue-flicking (RTF), there was a significant sex × trail type interaction (F_1,27_ = 5.21, P = 0.041), a marginal independent effect of sex (F_1,27_ = 3.43, P = 0.08), and no independent effect of trail type (F1_,27_ = 0.21, P = 0.65) (S5 Fig). Male RTF was higher than female RTF in the presence of scent trails (q = 4.00, P = 0.01) but not lipid trails (q = 0.28, P = 0.84), and male RTF was marginally higher for scent compared to lipid trails (q = 2.74, P = 0.07) (S5 Fig). No other differences were significant, and no other behavioral or temporal variables were significantly influenced by sex, trail type, or their interaction when comparing tegu performance between the two experiments.

## Discussion

Tegus successfully followed trails of isolated skin lipid extracts from conspecifics, and their behaviors were robust and generalized across the sexes. To our knowledge, this is the first proof-of-concept that tegu-derived cues, namely hexane-soluble skin lipids, elicit behavioral responses and active trailing in this invasive lizard. Further, tegus showed interest in but no preference for a novel and cheap source of neutral lipids (diluted peanut oil) in the bioassays.

Compared to our previous findings using tegu-created scent trails [25], skin lipids alone provide limited information that still facilitates successful conspecific tracking in tegus. Many species of lizards use multimodal signaling in mate choice where one sex assesses multiple communication modalities (e.g., behavioral displays, coloration, chemical cues) to discriminate among potential mates (e.g., [33, 34]). Chemical cues are ubiquitous sources of sexual signals in squamates and can function alone in mate assessment (e.g., [35]) or in combination with behavioral and/or visual signals (e.g., [36]). However, chemical cues are the primary if not singular modality enabling mate trailing across widely variable environments (reviewed in [7, 8]). Tegus in our study could still follow and choose conspecific and even opposite-sex skin lipid trails in the Y-maze, indicating that *Salvator merianae* is capable of fine-scale chemical discrimination that has previously been undocumented.

Chemical cues used in sexual communication in terrestrial vertebrates are often complex mixtures, and this is exemplified in squamate reptiles. Many snake species produce a series of long-chain lipids, such as squalene and methyl ketones, that serve as sex-specific signals driving male sex behavior (e.g., [12, 37]). These specific compounds naturally occur as a mixture on the surface of the skin, and they are part of the rich lipid matrix preventing transcutaneous evaporative water loss (reviewed in [38]). Similar lipid matrices are also produced by lizards, though many lizard species also have specialized glands (e.g., femoral pores) that produce lipid mixtures acting as potent sources of pheromones involved in reproductive isolation (e.g., [39]; reviewed in [8]). The pore secretions are also rich in proteins with unidentified but hypothesized functions in chemical signaling [40]. Tegus possess femoral glands, and the composition of the lipid mixture for males has been described [41]. While there is evidence that captivity alters the composition of the femoral pore secretions in male black and white tegus [42], the behavioral influences of those lipids are unknown as are the sex and seasonal variation of the secretions; such knowledge is valuable and would aid in understanding the chemoecology of this species in its invasive range. This species does demonstrate territoriality, which suggests that scent-marking using femoral pore secretions may be an important facet of their reproductive ecology [43]. For our study, the amount of lipids needed to conduct trailing experiments precluded femoral pores as the sole chemical cue source we could use in trailing experiments; often the amount of lipid produced per individual is too low to even enable small-scale chemical analysis (e.g., [44]). Instead, we chose shed skins as a lipid source because they are abundant and renewable. Most importantly, lipids from shed skins are scalable in management applications.

Current approaches for managing the Argentine tegu invasion in the Southeastern U.S. have focused on trapping and removal, primarily by baiting traps with chicken eggs which can be effective lures in both high and low density invasive populations (e.g., [45, 46]). Food cues are undoubtedly effective lures for trapping tegus and other large-bodied invasive lizards [23], but the cues attracting lizards to such traps are unknown. Further, trap deployment is often informed by survey data, including camera traps, and trap arrangements are clustered in suitable habitat, to maximize removal rates and increase return-on-investment [24, 47]. Trapping efforts have proven most effective for tegus when trap density is high and immigration low [24], but even in some of these high trap density areas, tegu removal efforts using current lures have been deemed ineffective at reducing tegu populations [24]. Tegus threaten many ecosystems beyond those of the Southeastern U.S., particularly biodiverse islands with endangered and endemic taxa [48]. Therefore, the development of effective control strategies has broad potential to mitigate the spread of tegus.

Recent observations on the spatial ecology of wild tegus in South Florida strongly suggest that conspecific scent trails drive movement patterns during the breeding season [49]. As such, leveraging the conspecific trailing behavior we have documented by way of developing lures that incite a similar trailing behavior is intuitive and logical. Lures that can be broadcast in a tegu’s territory or during immigration/emigration could increase trapping efficiency. Our results suggest that skin lipids could be deployed as scent nets or baited lines directing tegus toward high-density trapping locations. The high solubility of skin lipids in dispersible solvents such as hexane facilitates large-scale application of such trails [25, 31]. A subsequent phase of this work that could be fruitful would be to scale-up extraction of lipids and leverage captive tegus to test broader application in novel, open habitat (i.e., a large outdoor pen) where tegu behavior and movement patterns toward baited traps can be readily observed. Such a study would also allow estimation of the influence of scent trails alone on larger-scale movement patterns in tegus. The persistence of chemical signals in the environment, either naturally deposited or artificially applied, is an important consideration because of the strong, negative influence of temperature and humidity on such signals [50]. In at least one lizard species, chemical signals decayed rapidly at high environmental temperatures and lost their signaling potency [51]; thus, assessing the impact of temperature and/or time on the quality of tegu chemical trails would be an important and informative next step. Alternatively, shed skins may enhance trapping rates simply by being added alongside chicken egg baits as additional sources of attractive chemical cues. However, across any of the mentioned applications, we note that skin lipids may only be effective seasonally given that tegus respond differentially across the year to conspecific scent [25]. While the challenges of managing invasive reptiles are manifold, the results of our study strongly suggest that skin lipids are effective sources of conspecific information that influence mate searching behavior in Argentine black and white tegus.

## Supporting information

S1 Fig Tegu shed skin mass scales with extracted lipid mass. To source skin lipids for testing in behavioral trials, shed skins from Argentine black and white tegus were extracted in hexane. The efficiency of the extraction is determined by regressing extracted lipid mass on shed skin mass. Previous work demonstrated that this relationship is linear when both variables are log-transformed. The larger the mass of shed skin, the greater the predicted mass of extracted lipids.
(TIF)

S2 Fig Examples of tegu chemosensory behaviors observed in Y-maze trials. Tegus demonstrated several behaviors that were easily quantified from the trials, such as tongue-flicking, turns, and pauses.
(MP4)

S3 Fig Comparison of choice penalty scores. Choice penalty scores did not differ based on skin lipid trail type nor sex of the focal tegu. Choice penalty indicates the degree of non-target arm exploration (i.e., more negative scores indicate greater exploration of the unscented or non-target arm of the maze). Bars are means (+SEM; -95% C.I.).
(TIF)

S4 Fig Trailing times in the Y-maze. Tegus did not spend differential amounts of time trailing within the maze across the trial types. Bars are means (+SEM; -95% C.I.).
(TIF)

S5 Fig Chemosensory sampling behavior (rate of tongue-flicking; RTF) differed based on sex and trailing scenario. Rates of tongue-flicking were marginally lower for males (0.05 < P < 0.10) when only lipid trails were present in the Y-maze. Bars are means (+SEM; -95% C.I.).
(TIF)

S1 Table All behavioral data collected from the Y-maze skin lipid trail tests. This file contains the individual behaviors scored per tegu per trail setup (Male-only, Female-only, Male vs. female). The first tab contains the data collected and used in analyses, the second tab contains the skin lipid extract data, and the last tab contains the biometry data for the individual tegus used in the behavior trials.
(XLSX)
