# Supplementary figures and images for: Skin lipids alone enable conspecific tracking in an invasive reptile, the Argentine black and white tegu lizard (Salvator merianae)
Source: PLoS One. 2023 Oct 31;18(10):e0293591. doi: 10.1371/journal.pone.0293591 (PMC10617720; doi:10.1371/journal.pone.0293591)

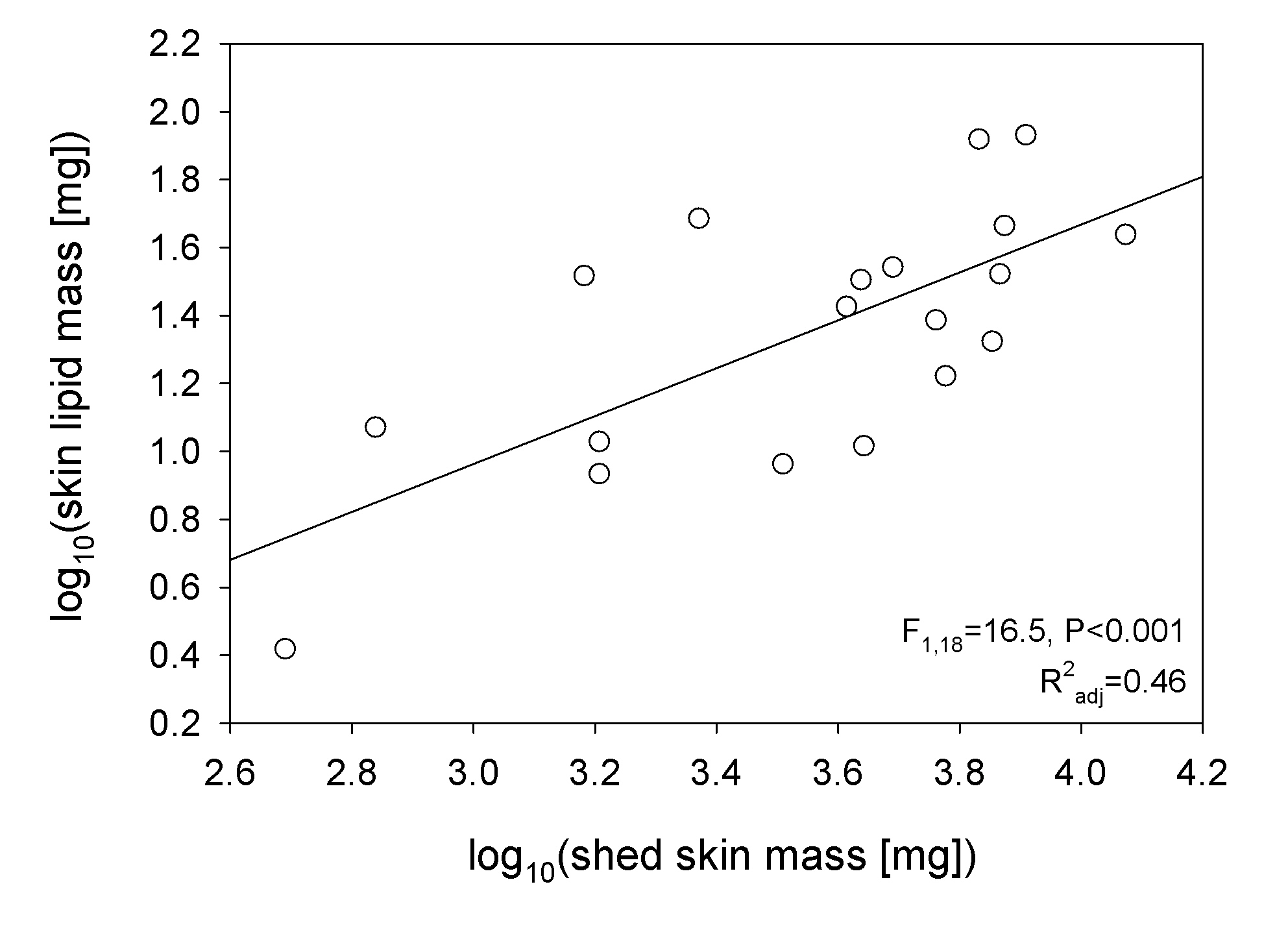

Supplement: S1 Fig — To source skin lipids for testing in behavioral trials, shed skins from Argentine black and white tegus were extracted in hexane. The efficiency of the extraction is determined by regressing extracted lipid mass on shed skin mass. Previous work demonstrated that this relationship is linear when both variables are log-transformed. The larger the mass of shed skin, the greater the predicted mass of extracted lipids. (TIF) [file pone.0293591.s001.tif]

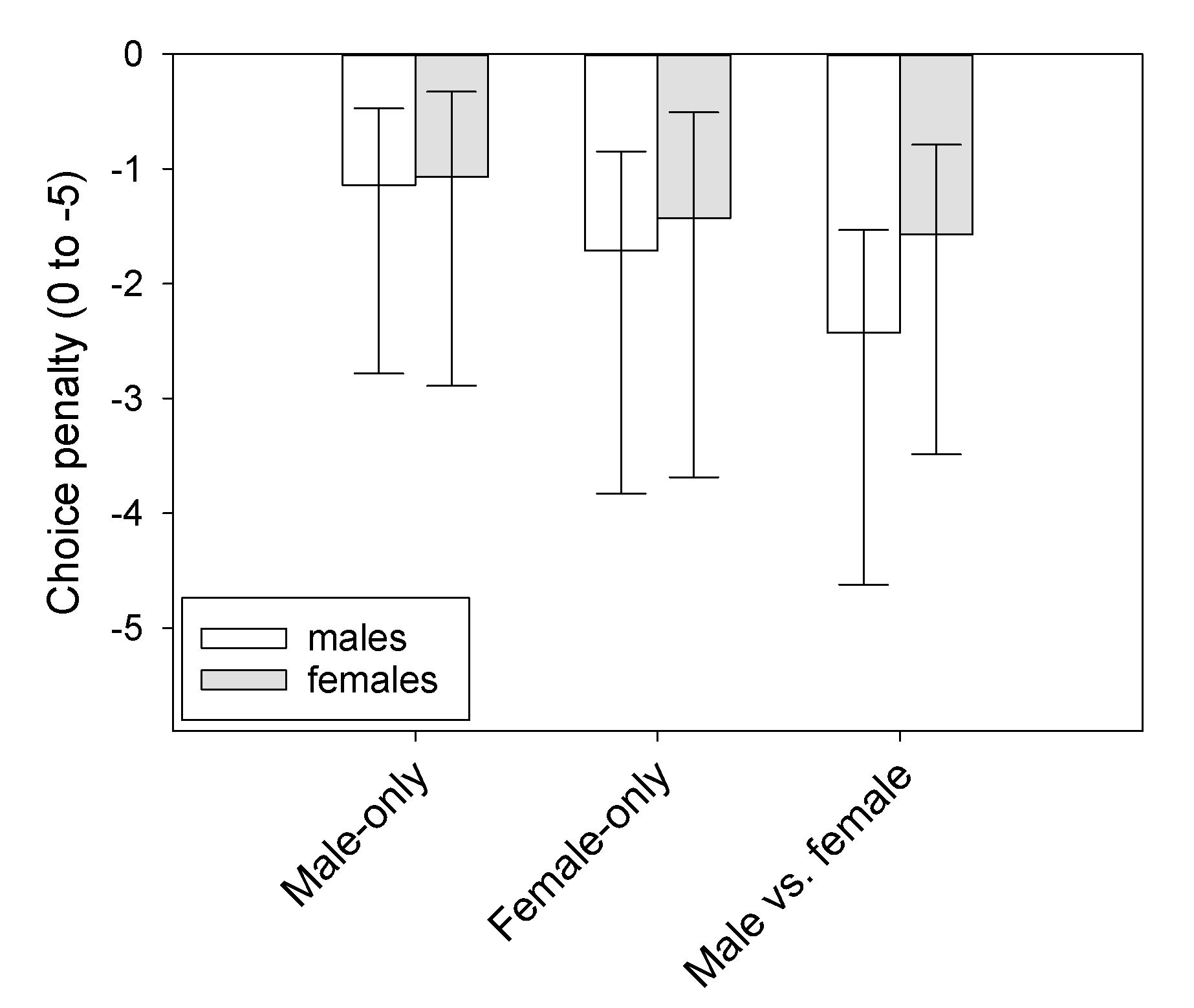

Supplement: S3 Fig — Choice penalty scores did not differ based on skin lipid trail type nor sex of the focal tegu. Choice penalty indicates the degree of non-target arm exploration (i.e., more negative scores indicate greater exploration of the unscented or non-target arm of the maze). Bars are means (+SEM; -95% C.I.). (TIF) [file pone.0293591.s003.tif]

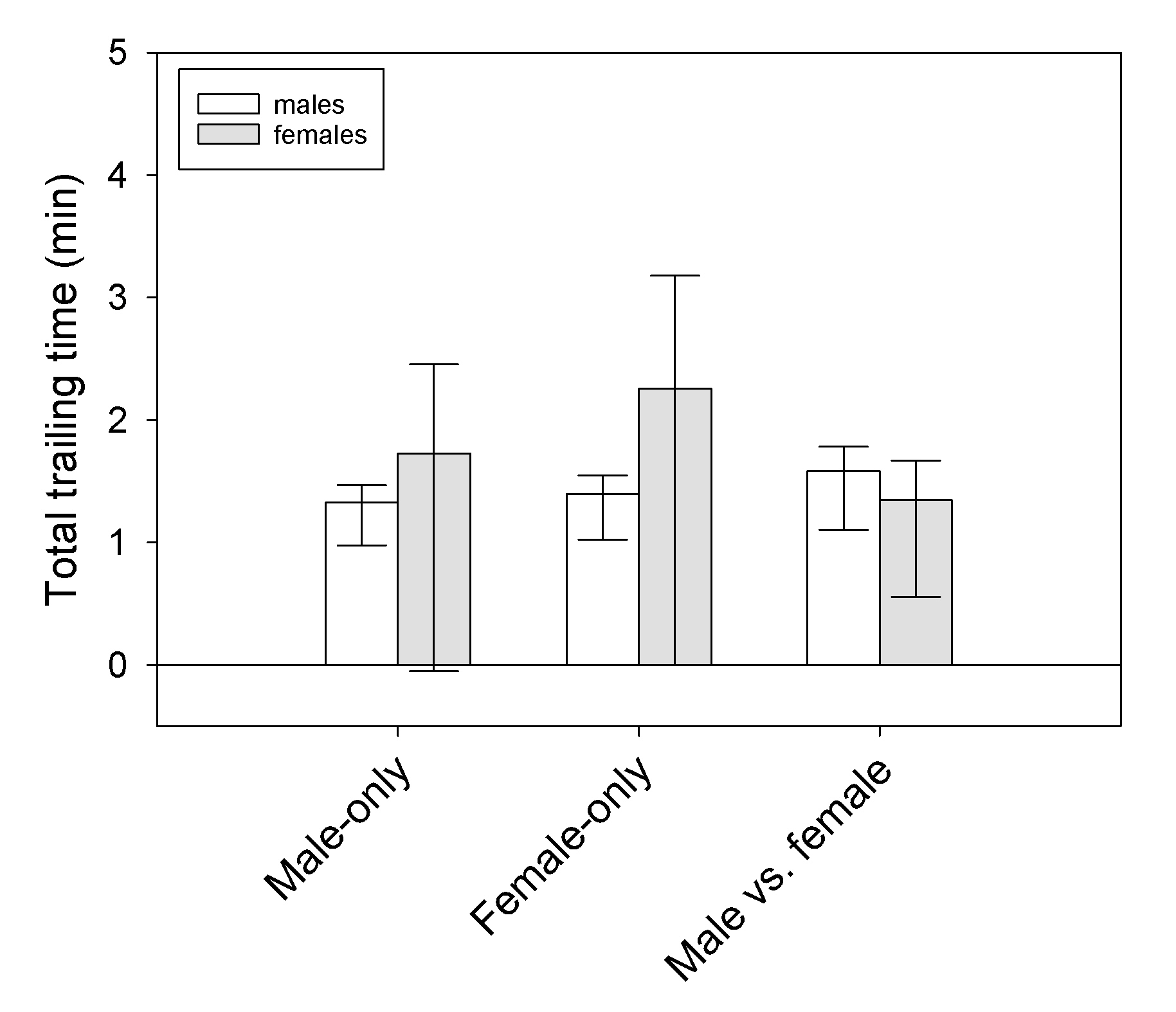

Supplement: S4 Fig — Tegus did not spend differential amounts of time trailing within the maze across the trial types. Bars are means (+SEM; -95% C.I.). (TIF) [file pone.0293591.s004.tif]

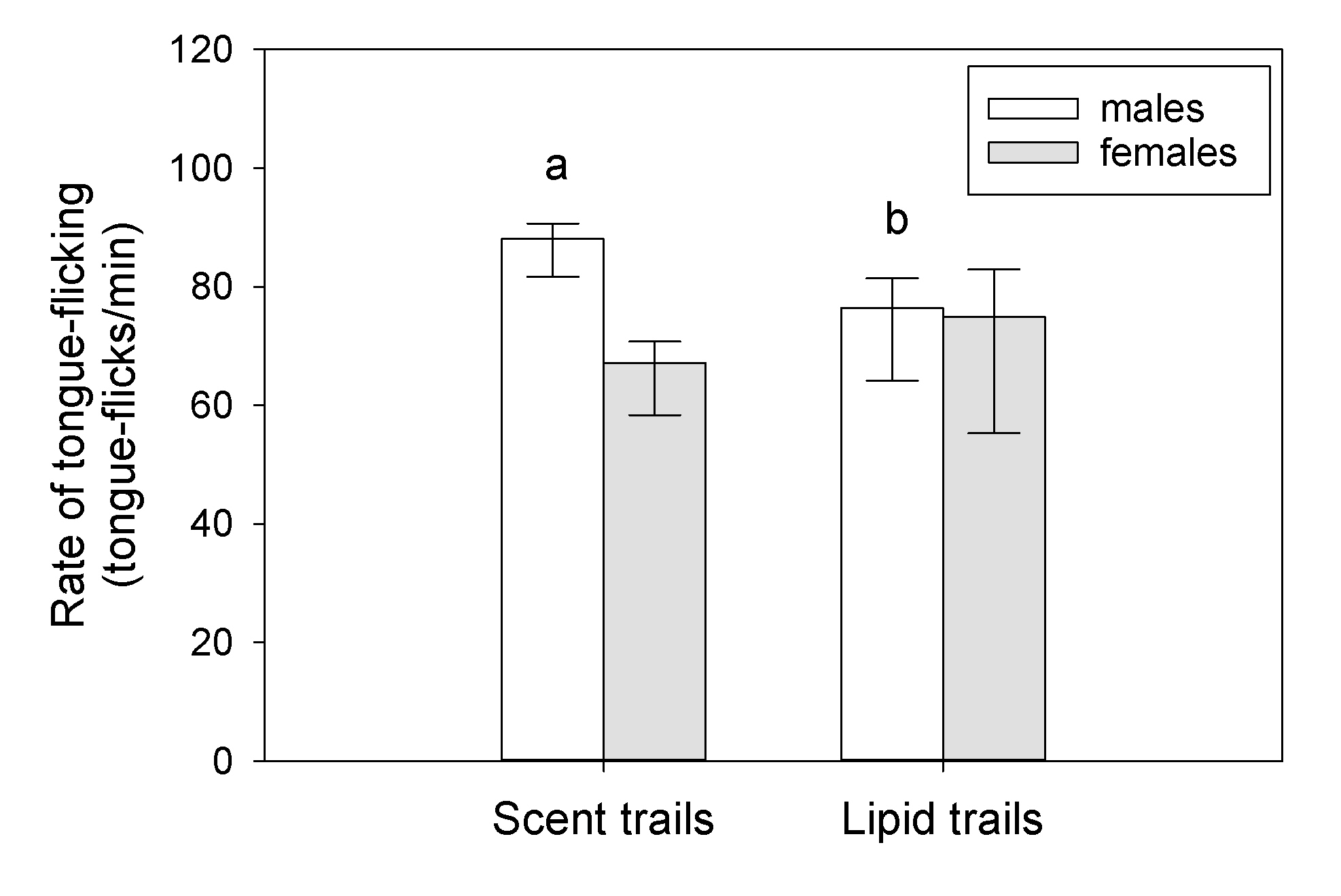

Supplement: S5 Fig — Rates of tongue-flicking were marginally lower for males (0.05 < P < 0.10) when only lipid trails were present in the Y-maze. Bars are means (+SEM; -95% C.I.). (TIF) [file pone.0293591.s005.tif]
